# Pseudo-Quantum Electrodynamics: 30 Years of Reduced QED

**DOI:** 10.3390/e26110925

**Published:** 2024-10-30

**Authors:** Eduardo C. Marino, Leandro O. Nascimento, Van Sérgio Alves, Danilo T. Alves

**Affiliations:** 1 Instituto de Física, Universidade Federal do Rio de Janeiro, Rio de Janeiro 21941-972, Brazil; marino@if.ufrj.br; 2 Faculdade de Física, Universidade Federal do Pará, Belém 66075-110, Brazil; lon@ufpa.br (L.O.N.); danilo@ufpa.br (D.T.A.)

**Keywords:** pseudo-quantum electrodynamics, reduced quantum electrodynamics

## Abstract

Charged quasiparticles, which are constrained to move on a plane, interact by means of electromagnetic (EM) fields which are not subject to this constraint, living, thus, in three-dimensional space. We have, consequently, a hybrid situation where the particles of a given system and the EM fields (through which they interact) live in different dimensions. Pseudo-Quantum Electrodynamics (PQED) is a U(1) gauge field theory that, despite being strictly formulated in two-dimensional space, precisely describes the real EM interaction of charged particles confined to a plane. PQED is completely different from QED(2 + 1), namely, Quantum Electrodynamics of a planar gauge field. It produces, for instance, the correct 1/r Coulomb potential between static charges, whereas QED(2 + 1) produces lnr potential. In spite of possessing a nonlocal Lagrangian, it has been shown that PQED preserves both causality and unitarity, as well as the Huygens principle. PQED has been applied successfully to describe the EM interaction of numerous systems containing charged particles constrained to move on a plane. Among these are p-electrons in graphene, silicene, and transition-metal dichalcogenides; systems exhibiting the Valley Quantum Hall Effect; systems inside cavities; and bosonization in (2 + 1)D. Here, we present a review article on PQED (also known as Reduced Quantum Electrodynamics).

## 1. Introduction

The electronic interaction between graphene and some two-dimensional materials, from first principles, involves the full electromagnetic interaction described by the minimal coupling of the electronic current to the U(1) electromagnetic gauge field. Incorporating this interaction into the model is challenging because electrons in such systems are confined to a plane, requiring a 2D description, while the electromagnetic field is inherently 3D. Using Maxwell electrodynamics in 2D would yield incorrect results, such as an electrostatic potential of lnr instead of the correct Coulomb potential of 1/r. To address this issue, a 2D U(1) gauge field theory was developed, which captures the complete physics of the 3D Maxwell theory within a 2D framework. This theory is known as Pseudo-Quantum Electrodynamics (PQED) [1], also called Reduced Quantum Electrodynamics [2] or Mixed-dimensional QED [3] (the reason why the name “Pseudo QED” seems preferable is because the gauge field $A_{\mu }$ (μ=0,1,2), which appears in Equation (1), is neither a reduction (projection) of the electromagnetic field $A_{\mu }$ (μ=0,1,2,3) nor it mixes with it in any sense; the gauge field in PQED lives completely in 2+1D and couples just to two-dimensional charged matter). It was initially derived in the static limit in Refs. [4,5]. PQED is particularly useful for systems where charged fermions are confined to geometries with spatial dimensions less than three, while the electromagnetic field propagates beyond the confining geometries. These systems are described by the standard 3D Maxwell equations with sources localized in lower dimensions. The PQED model is described by the following Lagrangian density:

(1) $$L_{\mathrm{PQED}}=\frac{1}{2}\frac{F_{\mu \nu }F^{\mu \nu }}{{\left(-□\right)}^{1/2}}+L_{D}+j^{\mu }A_{\mu }-\frac{\xi }{2}A_{\mu }\frac{\partial^{\mu }\partial^{\nu }}{{\left(-□\right)}^{1/2}}A_{\nu },$$

where $F_{\mu \nu }=\partial_{\mu }A_{\nu }-\partial_{\nu }A_{\mu }$, □ is the d’Alembertian operator, $L_{D}$ stands for the Dirac’s Lagrangian, and the last term corresponds to the gauge fixing term.

Despite the nonlocality of the Maxwell term in Equation (1), the theory satisfies causality [6], meets the Huygens principle, preserves unitarity [7], and presents scale invariance [8,9] and gauge invariance [1]. In addition, it reproduces the static Coulombian potential (∝1/r), instead of the peculiar logarithmic one from QED in 2+1 dimensions (∝lnr). Recently, PQED was used in the description of several electronic properties in 2D systems [10,11,12,13,14,15,16,17,18,19,20,21,22,23].

The paper is organized as follows: Unitarity, causality, and the Huygens principle are discussed in Section 2. The renormalization of the Fermi velocity in graphene is explored in Section 3. Section 4 examines the Quantum Valley Hall effect driven by interactions. The renormalization of the band gap in WSe_2_ and MoS_2_ is addressed in Section 5. The g-factor in graphene is analyzed in Section 6. Section 7 covers the energy spectrum and lifetime of excitons. In Section 8, we discuss bosonization and other versions of PQED. Cavity PQED and *N*-Layers PQED are presented in Section 9. Final remarks are found in Section 10.

## 2. Unitarity, Causality, Huygens Principle, and Gauge Invariance

One of the most unexpected feature of PQED, in comparison to standard models in Quantum Field Theory (QFT), is the presence of the pseudo-differential operator ${\left(-□\right)}^{-1/2}$ in its gauge field action. Although we may argue that quantum corrections are calculated in the momentum space, such that we may perform ${\left(-□\right)}^{-1/2}\to {\left(k^{2}\right)}^{1/2}$ to calculate them, an important question remain open: is the whole model consistent with quantum physics? The answer is yes, as it has been shown in Ref. [7]. Next, let us discuss the main idea behind such result. Having in mind the probabilistic interpretation, the first step is to check whether the theory respects unitarity.

The unitarity of the *S*-matrix ensures that the sum of the probabilities is conserved and hence may be kept equal to one everywhere. This, however, may be translated into an equation (the optical theorem) for the nontrivial part of *S*, which is called the transfer matrix *T*. In QFT, the expectation value of *T* between asymptotic states is, essentially, proportional to the propagator of the basic field plus momentum conservation. Therefore, we have a powerful tool for checking unitarity in the model, which combines the optical theorem and the propagator. It turns out that the Maxwell propagator in (2+1)D is connected to the propagator of PQED by a Fourier transform, i.e., they are dual solutions of the optical theorem, where one is written in the coordinate spacetime, while the other is the solution in the energy–momentum space. This is the main reason why PQED is consistent with the probabilistic interpretation, since it only emulates QED3 in the coordinate spacetime. From a more physical point of view, it is also reasonable because PQED describes the same electronic dynamics as QED4, except for the fact that we neglect a spatial component of the matter current, usually by taking $j_{z}=0$. This, however, should note break any conservation law. The static regime of PQED is even more clearly consistent with quantum physics, as it represents a set of two-dimensional Dirac electrons interacting through the Coulomb potential.

While unitarity makes the theory consistent with quantum physics, a QFT model also has to be in agreement with special relativity, which is corroborated by its causality. In QFT, this is usually stated by requiring that the commutator between the field and its canonical conjugated momentum vanishes outside the light cone. This quantity defines a Green function, called the Pauli–Jordan function, which is normally calculated to verify the causality. In Ref. [6], it has been shown that PQED keeps this feature similar to QED4 and QED3. When considering the classical propagation of light waves, an important feature is the Huygens principle, which claims that the signals travel at light velocity for any source, even for a secondary source such as a slit. In field theory, this may be ensured by proving that the retarded Green function has its support on the surface of the light cone, i.e., it vanishes for space-like coordinate vectors. Obviously, the Maxwell theory in (3+1)D satisfies this condition. However, in (2+1)D, the Maxwell propagator violates the Huygens principle. Surprisingly, it has been shown that, due to the ${\left(-□\right)}^{-1/2}$ operator, the gauge field in PQED satisfies the Huygens principle [24].

Gauge invariance occurs in PQED [1] similarly to the Stueckelberg field (see Refs. [25,26]). In this case, one can show that a Maxwell-like term, such as $m^{2}F_{\mu \nu }^{2}/\left(□\right)$ (after integrating out the scalar field of the Stueckelberg model), results in a mass term for the photon without breaking gauge invariance. Since $F_{\mu \nu }^{2}$ is gauge-invariant (regardless of the □), this symmetry is preserved. This is advantageous compared with the standard massive model known as Proca-QED, where gauge invariance is broken. This point is discussed in detail, for instance, in Ref. [27]. In PQED, we also have an action depending solely on $F_{\mu \nu }^{2}$. Furthermore, charge conservation holds in (2+1)D when the *z*-component of the matter current 4-vector vanishes, i.e., $j_{z}=0$.

## 3. Renormalization of Fermi Velocity in Graphene

Although PQED was first derived as a QFT-model, its main constraint, that is, to confine particles in a two-dimensional space, is usually realized in condensed matter systems [1], in particular, graphene and other two-dimensional materials with a honeycomb lattice. In these systems, the charge carriers (also called quasiparticles) behave as Dirac particles with a typical velocity called Fermi velocity $v_{F}$. For graphene, these quasiparticles are massless, yielding the notorious Dirac cones in the energy-band spectrum, and $v_{F}\approx c/300$ is a small fraction of the light velocity *c* [28]. The description of the electromagnetic interaction between these carriers is a straightforward application of PQED.

In 1994, before the experimental realization of graphene, it was first shown that $v_{F}$ is renormalized by quantum effects of the Coulomb interaction between electrons confined to a plane. The main result establishes that at very low density of charge carriers (n→0), the Fermi velocity increases as a function $v_{F}\left(n\right)\propto v_{F}\left[1-\alpha \ln\left(n/n_{0}\right)\right]\to \infty$, where α≈2.2 is the effective fine-structure constant of the material [29]. This result suffices to show the relevance of the interactions. Nevertheless, because it is the static limit of PQED, it yields an infinite velocity in the zero-doping regime instead of the correct relativistic limit, where $v_{F}\left(n\right)\to c$ at n→0, as discussed in Ref. [30].

In 2023, the temperature effects were calculated into the renormalized Fermi velocity. In the low-temperature limit, the renormalization of the Fermi velocity is described by $v_{F}\left(p,T\right)-v_{F}\left(p,0\right)\approx -B_{\alpha }{\left(k_{B}T/p\right)}^{3}$, where *T* is the temperature of the thermal bath, $B_{\alpha }$ is a known function of α, $k_{B}$ is the Boltzmann constant, and *p* is an energy scale. For T=0, *p* is exactly the Fermi energy, and this, on the other hand, is written in terms of *n*. This result is expected to be accurate for temperatures below $10^{2}$K. However, the full dependence on the temperature has also been shown in Ref. [31]. In particular, for higher temperatures ($k_{B}T\gg \Lambda \approx 1$ eV), the quantum corrections are strongly suppressed, and the Femi velocity remains equal to its bare value. The parameter Λ is the energy cutoff of the model and provides an upper energy limit for the validity of the Dirac approximation to the matter field in these materials.

## 4. Quantum Valley Hall Effect Driven by Interactions

The band theory of solids has been through a recent change after the discovery of topological insulators [28]. The well-established classification of metals and insulators has been modified in order to accommodate materials that are insulators in the bulk but conductors at their edges, which are known as Chern insulators in (2+1)D. One of the most important results is the bulk–boundary correspondence, which establishes that the number of energy levels crossing the Fermi energy is equal to the conducting modes at the edge of the material. Hence, it is enough to calculate the spectrum in the bulk for obtaining the behavior at the edge of the material. On the other hand, the manipulation of the valleys (the two inequivalent Dirac cones *K* and $K^{\prime }$) in the honeycomb lattice is expected to generate a new area of applications for two-dimensional materials called valleytronics [28].

In 2015, it was already known that the Coulomb interaction is relevant in graphene due to both theoretical and experimental findings about Fermi velocity renormalization [29,32]. Within this interacting regime, the PQED formalism is a useful tool for investigating interaction-driven effects, in particular, the renormalization of the band-gap spectrum in the bulk of two-dimensional materials, such as graphene. In this case, Equation (1) provides a set of quantized energy levels whenever the fine-structure constant of graphene is larger than a critical parameter (whose expected value is quite close to one) and for temperatures small enough such that the energy levels do not vanish due to thermal activation (below 2 K) [14]. It turns out that each energy level in the bulk leads to a conducting edge mode, each one carrying a quantum of conductivity given by $e^{2}/h$, where *e* is the fundamental electric charge and *h* is the Planck constant. The electric current, nevertheless, depends on the sign of the mass of the quasiparticle. Here, the sign of the mass is the sign of the $\bar{\psi }\psi$-term, where ψ describes the Dirac field, in the effective low-energy action of the quasiparticle, which is different for *K* and $K^{\prime }$. Because the valleys are connected by a time-reversal symmetry, it follows that they necessarily have opposite masses. Hence, they generate counter-propagating currents, which are the main signature of the interaction-induced Quantum Valley Hall effect in graphene at zero magnetic field. The existence of the Quantum Valley Hall effect has been discussed in the literature; in particular, some experimental findings have been reported in graphene superlattices [33] and monolayers of SiC [34].

## 5. Renormalization of Band Gap in WSe_2_ and MoS_2_

There exist other two-dimensional materials [35] beyond graphene that have attracted great attention due to their peculiar electronic properties, in particular, the monolayers of transition-metal dichalcogenides, such as WSe_2_ [36] and MoS_2_ [37]. Similarly to graphene, they also have a honeycomb lattice; however, due to the breaking of sublattice symmetry, a nonzero band gap of the order 1–2 eV emerges in their spectrum [30]. This quantity is readily described by a mass term in the Dirac equation for quasiparticles at low energies [38].

In 2020, the authors of Ref. [30] showed that the band gap is a function of the carrier density *n*, namely, $m\left(n\right)=m_{0}{\left(n/n_{0}\right)}^{C_{\alpha }}$, where $m_{0}$ is some particular value of the gap at density $n=n_{0}$ and $C_{\alpha }$ is a known function of α. For WSe_2_, the band gap decreases by an amount of approximately 400 meV from its bare value (close to 2.2 eV), when the carrier density changes within the interval of $n\in \left[1.0,15\right]\times 10^{12}$ cm^−2^. Because the substrate changes α, the coupling constant is expected to be in the range α∈[0.97,1.22] for the two substrates that have been considered for comparison with the experimental data. The theoretical description of m(n) has been shown to be quite successful when compared with the experimental data. In 2023, this result was generalized to the case where a thermal bath of temperature *T* is considered [31]. Within that work, the main conclusion is that at low temperatures, the renormalized mass is a decreasing linear function of *T*. However, for very large temperatures, the mass remains at its bare value, as expected.

Although PQED has some relevant applications in two-dimensional materials, it is fairly easy to conclude that it is not the full model for describing interactions in a graphene-like system. Indeed, quasiparticles in a crystal may undergo several interactions, such as impurities, disorder, and mechanical vibrations, just to cite a few. These sets of effects are usually considered as microscopic interactions within an effective low-energy model, and they may be described by four-fermion interactions; see Refs. [39,40]. In 2021, the effects of these four-fermion interactions were considered in Ref. [41] with an extra coupling constant λ beyond α. The competition between these constants shows that we may have an increase in the renormalized band gap, as a function of *n*, whether $\lambda >\lambda_{c}$, where $\lambda_{c}\in \left[0.26,0.66\right]$, depending on the type of four-fermion interaction we consider in the effective model.

## 6. The g-Factor in Graphene

The gyromagnetic factor (g-factor) of the electron in vacuum relates its magnetic dipole moment and spin. Without quantum effects, it is equal to g=2, which is obtained from the Dirac theory. When considering quantum correction at one-loop approximation, calculated from QED4, we find $g=2+\alpha_{\mathrm{QED}}/2\pi$, where $\alpha_{\mathrm{QED}}\approx 1/137$ is the standard fine-structure constant. This result already is in good agreement with the experimental data; however, including higher-order corrections, we have one of the best theoretical predictions, as it is discussed in Ref. [42].

There have been some experimental findings reporting the g-factor for the quasiparticles in graphene in Refs. [43,44], where it is shown that the *g*-factor is dependent on both the substrate and the carrier density *n*. The substrate is another material on top of which to place graphene; hence, it changes the dielectric constant ϵ, which is relevant for calculating the value of $\alpha \propto e^{2}/\epsilon v_{F}\left(n\right)$ and therefore changes the quantum correction. The dependence on *n* is also relevant because it renormalizes $v_{F}\left(n\right)$, making another contribution to the *g*-factor. In 2017, using the PQED formalism, the authors in Ref. [18] calculated the one-loop correction, at zero temperature, to the *g*-factor in graphene and described its dependence on *n* with good agreement with the previous experimental findings. These theoretical results have been used for describing the g-factor in graphene placed above SiO_2_ and SiC with a magnetic field ranging from 11*T* to 14*T*. Finally, the theoretical dielectric constant found is large, with ϵ∈[2,5], which opens an interesting discussion regarding screening effects in two-dimensional materials.

It is worth to mention that within the PQED approach, the quantum correction to the *g*-factor in graphene, namely, Δg, is drastically modified whether we consider the static or ultrarelativistic limit. The former is identified as the limit where the velocity is small enough ($v_{F}\left(n\right)\ll c$), such that it is enough to consider only the Coulomb interaction. The latter is defined as the regime where $v_{F}\left(n\right)\to c$; hence, one must consider the whole quantum-electrodynamical approach, whose interaction is given by $ej_{\mu }A^{\mu }$, where $j_{\mu }$ is the matter current. Indeed, in the static limit, Δg=α/4, whereas in the ultrarelativistic limit, we have Δg=−4α/3π. For an arbitrary velocity, Δg is a function of $v_{F}\left(n\right)/c$. These results, therefore, also direct our attention towards considering the dynamical regime, where no assumption is made about the full interaction, which is the most general case.

## 7. The Energy Spectrum and Lifetime of Excitons

The study of bound states is of fundamental importance, with applications from atomic to solid physics. For transition-metal dichalcogenides, there have been several experimental findings regarding the measurements of excitons, a bounded pair made of one electron and a hole; see Refs. [45,46,47]. The presence of these pairs changes the optical properties of the material, since an incident photon with energy equal to the binding energy $E_{b}$ of the pair is not reflected. Furthermore, the electron–hole pair has a finite lifetime τ and may decay into a photon. These parameters, $E_{b}$ and τ, constitute the main data for describing an exciton. For WS_2_, WSe_2_, and MoS_2_, some experimental data have shown that $E_{b}\in \left[30,400\right]$ meV, whereas τ∈[2,4] ps in these materials [48].

The simplest solution for calculating $E_{b}$ for two-dimensional excitons would be to solve the hydrogenic-like Schrödinger equation in 2D, obtain the energy levels, and translate them as the binding energy of the exciton. This, however, leads to a result that is not in agreement with the experimental data. However, the Keldysh potential, within a similar approach, has been shown to offer a much better result, as discussed in Ref. [46]. The Keldysh potential has been originally derived in Ref. [49] for describing the electromagnetic interaction. This potential is equal to the Coulomb interaction at very short distances and quickly goes to zero at large distances.

In 2018, the PQED formalism was used to describe the formation of excitons in these materials [48]. Interesting, it reproduces the Keldysh potential when considering quantum corrections to the Coulomb potential, generated by a massive Dirac particle. The binding energies, on the other hand, are calculated by taking the lowest-order solution of the Bethe–Salpeter equation, which is consistent with the full electron propagator, given by the Schwinger–Dyson equations. The energy spectra of the excitons are described by mid-gap states, given by $|E_{b}|=\Lambda e^{-X_{n}\left(\alpha -\alpha_{c}\right)}$, where Λ is the cutoff of the model and $X_{n}\left(\alpha -\alpha_{c}\right)$ is a real-valued function where $\alpha >\alpha_{c}=2/\pi$. For $\alpha <\alpha_{c}$, the interaction is not strong enough to produce the bounded pair. The number *n* is an integer that has been used to describe the five different binding energies observed in the measurements. The exponential decay of the binding energies, which is quite similar to a Miransky scaling law, has been shown to be much more promising than the hydrogenic-like approach. Furthermore, the calculated lifetime of the excitons is also in agreement with the experimental data [48].

## 8. Bosonization and Other Versions of PQED

Certainly, the most striking initial feature of PQED is the presence of the pseudo-differential operator in its gauge field. The history of the square root of the D’Alembert operator ${\left(-□\right)}^{-1/2}$, however, did not actually start with PQED. In 1990, it appeared in Ref. [4] in a model describing the superconductivity driven by a topological mass. In 1991, it was used for realizing the bosonization of massless Dirac fermions in (2+1)D [50]. Finally, in 1993, PQED was derived from a dimensional reduction of QED4. In the same work, the dynamics of the Chern–Simons action was discussed in (2+1)D [1]. It is worth to mention that in 2001, PQED was renamed Reduced Quantum Electrodynamics (QED), which is the name chosen by some authors [2,51,52,53,54]. The simplest classification of particles, considering the spin–statistics theorem, is between fermions and bosons. When considering QFT, it is possible to consider a process called bosonization, where we obtain a bosonic action that reproduces the same current–current correlation functions as the original fermionic action [38]. In 2021, it was shown that the bosonized version of the massive Dirac theory in (2+1)D may be built from PQED and a pseudo-Chern–Simons term, which is essentially the usual Chern–Simons term with a kernel given by ${\left(-□\right)}^{-1/2}$. Surprisingly, this action yields a mass for the gauge field, and its static limit admits the generation of bounded states [55]. In 2018, dimensional reduction was also applied to QED4 in the presence of a spontaneous symmetry breaking, which yields a fully (2+1)D theory describing the Yukawa interaction between the particles confined in the two-dimensional space [26]. This model also has a pseudo-differential operator, similarly to PQED.

## 9. Cavity PQED and N-Layers PQED

When the dimensional reduction from QED4 to PQED takes into account the effects of boundary conditions imposed on the electromagnetic field in (3+1)D, the influence of such external conditions is carried into PQED, producing the so-called Cavity PQED [56,57], a term suggested in analogy with Cavity QED, a branch of QED which investigates how the boundary conditions imposed by the environment influence the radiative properties of atomic systems. In the context of the Cavity PQED, some results have been obtained, as discussed next.

In Ref. [56], it was shown that the logarithmic renormalization of the Fermi velocity, found in Ref. [58], is inhibited by the presence of a grounded, perfectly conducting surface. This inhibition occurs because the effective interaction of the electrons in a graphene sheet is affected (reduced) by the presence of the plate, changing the photon propagator and, consequently, the electron self-energy [56]. For small distances between the graphene sheet and the plate, for instance, ≈1/Λ or ≈10/Λ (Λ is of the order of the inverse lattice parameter of graphene), the results found in Ref. [56] show a significant inhibition of the renormalized Fermi velocity. However, even for larger distances in comparison to the graphene lattice parameter, for instance, ≈100/Λ or $10^{5}/\Lambda$, the renormalized Fermi velocity will be still inhibited. The inhibition becomes more significant for lower values of the external momentum [56].

In Ref. [57], it was investigated how the inhibition of the renormalization of the Fermi velocity behaves when one brings two grounded, perfectly conducting plates near a suspended graphene sheet (which is between these plates). In this case, the inhibition is amplified in comparison to the case of a single plate found in Ref. [56], but it is not a mere addition of the inhibition induced by each single plate separately, with the presence of a term depending on the interaction between the plates. Moreover, it was shown that the results could have consequences in some transport properties of graphene, since the difference between the optical conductivity and the universal conductivity in graphene depends inversely on the renormalized Fermi velocity; therefore, the inhibition of the renormalized Fermi velocity could lead to an increase in the optical conductivity [57].

In Ref. [59], the influence of a grounded, perfectly conducting surface on the photon self-energy of a (2+1)D system of massless Dirac fermions and on the longitudinal and optical conductivity of such system was investigated [59]. By computing the temporal component of the polarization tensor up to the 2-loop perturbation order for the (2+1)D system in the presence of a grounded, perfectly conducting plate, the authors obtained the longitudinal and optical conductivity, according to the Kubo formula. The authors showed that the longitudinal conductivity increases as we bring the conducting surface closer to the (2+1)D system, and in the optical limit, the conductivity can increase or decrease, depending on the position of the conducting plate [59].

In Ref. [60], the authors investigated, in the context of Cavity PQED and within the framework of the random phase approximation, the effect of an interface (between two nondispersive semi-infinite dielectrics, with dielectric constants $\epsilon_{1}$ and $\epsilon_{2}$) on the renormalization group functions in a two-dimensional Dirac-like system located at a distance $z_{0}$ from the interface. The results predict the behavior of the renormalized mass, Fermi velocity, and the anomalous dimension of the fermion field under the influence of such interface. In the limits $\epsilon_{1}=\epsilon_{2}$ and $z_{0}=0$, the results reflect the corresponding ones found in Ref. [30], according to which the renormalized mass and Fermi velocity depend on Λ but the renormalized anomalous dimension does not. For $\epsilon_{1}\neq \epsilon_{2}$ and $z_{0}\neq 0$, the formula shown in Ref. [60] introduces explicit dependence on Λ in the renormalized anomalous dimension, which means that in the presence of an interface, the anomalous dimension of electrons is dependent on the density of states (in Ref. [30], the anomalous dimension for a two-dimensional material placed on a single substrate does not exhibit such dependence). In the limit $\epsilon_{2}\gg \epsilon_{1}=1$, the result in Ref. [60] reflects the corresponding one found in Ref. [56], which predicts the inhibition of the renormalization of the Fermi velocity in a graphene sheet in the presence of a grounded, perfectly conducting surface, and in the limit $z_{0}\to \infty$, it reflects the renormalization of the Fermi velocity in graphene found in Ref. [61].

In Ref. [62], the dimensional reduction proposed by Marino was considered [1], but by incorporating the boundary conditions effects imposed by the presence of a partially reflective surface to the electromagnetic field, obtaining an effective Lagrangian for Cavity PQED, which enables, for instance, the calculation of the Fermi velocity renormalization for an electron in a graphene sheet near a conducting plate beyond the static interaction regime of the theory. In Ref. [63], the formalism of PQED was extend to the case of heterostructures composed of *N* layers, and the gap generation for massless Dirac fermions confined to two equivalent planes was investigated.

## 10. Final Remarks

The PQED model has proven to be a powerful and versatile (2+1)D field theory for describing and predicting various properties of two-dimensional materials where Dirac fermions are confined to a plane. Throughout this work, we have explored several key aspects of the PQED framework and its applicability to contemporary problems in condensed matter physics, particularly in the context of two-dimensional materials like graphene, WSe_2_, and MoS_2_.

The discussion began with an examination of fundamental concepts, such as unitarity, causality, and the Huygens principle, which are foundational to ensuring the consistency and physical relevance of any field theory. These principles were shown to hold within the PQED model, affirming its robustness. Subsequent sections addressed the renormalization of the Fermi velocity in graphene, a crucial parameter that influences the electronic properties of this material. The PQED model effectively captures the renormalization effects, offering insights into the interaction-driven modifications of the Fermi velocity. We also explored the Quantum Valley Hall effect, driven by electron–electron interactions. This phenomenon, which is of great interest in the study of topological phases of matter, was effectively described within the PQED framework, demonstrating the model’s capability to address interaction-driven topological effects. In addition, the renormalization of the band gap in two-dimensional materials such as WSe_2_ and MoS_2_ was investigated. The PQED model provides a comprehensive understanding of how interactions can modify the electronic band structure, highlighting its utility in studying band-gap engineering in 2D materials. The study further delved into the analysis of the g-factor in graphene, the energy spectrum, and the lifetime of excitons. These topics are pivotal to understanding the optical and magnetic properties of 2D materials, and the PQED model was shown to be an effective tool for predicting and explaining these phenomena. Moreover, the versatility of the PQED model was demonstrated through its application to bosonization and various other versions of PQED, as well as its extension to Cavity PQED and N-Layers PQED. These extensions illustrate the model’s adaptability and its potential to address a wide range of physical scenarios in two-dimensional systems.

In conclusion, the PQED model proves to be an effective theoretical framework for investigating and predicting the behavior of two-dimensional materials, particularly in scenarios where electromagnetic interactions play a significant role. Its success in describing unitarity, causality, velocity renormalization, topological effects, band-gap renormalization, and exciton dynamics, among others, underscores its importance as a tool in condensed matter physics. As research on 2D materials advances, the PQED model will remain a key asset in unraveling the complex interactions and phenomena that define these systems. An important avenue for future researches is the investigation of PQED in the presence of an external magnetic field, as this approach appears to be particularly relevant for understanding the Quantum Hall effect and the emergence of topological phases of matter.

## Data Availability

Not applicable.

## References

[1] Marino E. (1993). Quantum electrodynamics of particles on a plane and the Chern-Simons theory. Nucl. Phys. B.

[2] Gorbar E.V., Gusynin V.P., Miransky V.A. (2001). Dynamical chiral symmetry breaking on a brane in reduced QED. Phys. Rev. D.

[3] Kotikov A.V., Teber S. (2014). Two-loop fermion self-energy in reduced quantum electrodynamics and application to the ultrarelativistic limit of graphene. Phys. Rev. D.

[4] Kovner A., Rosenstein B. (1990). Kosterlitz-Thouless mechanism of two-dimensional superconductivity. Phys. Rev. B.

[5] Dorey N., Mavromatos N. (1992). QED3 and two-dimensional superconductivity without parity violation. Nucl. Phys. B.

[6] do Amaral R.L.P.G., Marino E.C. (1992). Canonical quantization of theories containing fractional powers of the d’Alembertian operator. J. Phys. A: Math. Gen..

[7] Marino E.C., Nascimento L.O., Alves V.S., Morais Smith C. (2014). Unitarity of theories containing fractional powers of the d’Alembertian operator. Phys. Rev. D.

[8] Dudal D., Mizher A.J., Pais P. (2019). Exact quantum scale invariance of three-dimensional reduced QED theories. Phys. Rev. D.

[9] Heydeman M., Jepsen C.B., Ji Z., Yarom A. (2020). Renormalization and conformal invariance of non-local quantum electrodynamics. J. High Energy Phys..

[10] Alves V.S., Elias W.S., Nascimento L.O., Juričić V., Peña F. (2013). Chiral symmetry breaking in the pseudo-quantum electrodynamics. Phys. Rev. D.

[11] Teber S., Kotikov A.V. (2014). Interaction corrections to the minimal conductivity of graphene via dimensional regularization. Europhys. Lett..

[12] Barnes E., Hwang E.H., Throckmorton R.E., Das Sarma S. (2014). Effective field theory, three-loop perturbative expansion, and their experimental implications in graphene many-body effects. Phys. Rev. B.

[13] Nascimento L.O., Alves V.S., Peña F., Smith C.M., Marino E.C. (2015). Chiral-symmetry breaking in pseudoquantum electrodynamics at finite temperature. Phys. Rev. D.

[14] Marino E.C., Nascimento L.O., Alves V.S., Smith C.M. (2015). Interaction Induced Quantum Valley Hall Effect in Graphene. Phys. Rev. X.

[15] Menezes N., Alves V.S., Smith C.M. (2016). The influence of a weak magnetic field in the Renormalization-Group functions of (2+1)-dimensional Dirac systems. Eur. Phys. J. B.

[16] Kotikov A.V., Teber S. (2016). Critical behavior of reduced *QED*_4,3_ and dynamical fermion gap generation in graphene. Phys. Rev. D.

[17] Alves V.S., Junior R.O.C., Marino E.C., Nascimento L.O. (2017). Dynamical mass generation in pseudoquantum electrodynamics with four-fermion interactions. Phys. Rev. D.

[18] Menezes N., Alves V.S., Marino E.C., Nascimento L., Nascimento L.O., Morais Smith C. (2017). Spin *g*-factor due to electronic interactions in graphene. Phys. Rev. B.

[19] Magalhães G.C., Alves V.S., Marino E.C., Nascimento L.O. (2020). Pseudo quantum electrodynamics and Chern-Simons theory coupled to two-dimensional electrons. Phys. Rev. D.

[20] Olivares J.A.C., Albino L., Mizher A.J., Raya A. (2020). Influence of a Chern-Simons term in the dynamical fermion masses in reduced or pseudo QED. Phys. Rev. D.

[21] Ozela R.F., Alves V.S., Magalhães G.C., Nascimento L.O. (2022). Effects of the pseudo-Chern-Simons action for strongly correlated electrons in a plane. Phys. Rev. D.

[22] Albino L., Bashir A., Mizher A.J., Raya A. (2022). Electron-photon vertex and dynamical chiral symmetry breaking in reduced QED: An advanced study of gauge invariance. Phys. Rev. D.

[23] Casimiro Olivares J.A., Mizher A.J., Raya A. (2022). Non-perturbative field theoretical aspects of graphene and related systems. Rev. Mex. Física.

[24] Bollini C., Giambiagi J. (1993). Arbitrary powers of D’Alembertians and the Huygens’ principle. J. Math. Phys..

[25] Ruegg H., Ruiz-Altaba M. (2004). The Stueckelberg field. Int. J. Mod. Phys. A.

[26] Alves V.S., Macrì T., Magalhães G.C., Marino E.C., Nascimento L.O. (2018). Two-dimensional Yukawa interactions from nonlocal Proca quantum electrodynamics. Phys. Rev. D.

[27] Schwartz M.D. (2014). Quantum Field Theory and the Standard Model.

[28] Bernevig B.A. (2013). Topological Insulators and Topological Superconductors.

[29] González J., Guinea F., Vozmediano M.A.H. (1994). Non-Fermi liquid behavior of electrons in the half-filled honeycomb lattice (A renormalization group approach). Nucl. Phys. B.

[30] Fernández L., Alves V.S., Nascimento L.O., Peña F., Gomes M., Marino E.C. (2020). Renormalization of the band gap in 2D materials through the competition between electromagnetic and four-fermion interactions in large *N* expansion. Phys. Rev. D.

[31] Bezerra N., Alves V.S., Nascimento L.O., Fernández L. (2023). Effects of the two-dimensional Coulomb interaction in both Fermi velocity and energy gap for Dirac-like electrons at finite temperature. Phys. Rev. D.

[32] Elias D.C., Gorbachev R.V., Mayorov A.S., Morozov S.V., Zhukov A.A., Blake P., Ponomarenko L.A., Grigorieva I.V., Novoselov K.S., Guinea F. (2011). Dirac cones reshaped by interaction effects in suspended graphene. Nat. Phys..

[33] Komatsu K., Morita Y., Watanabe E., Tsuya D., Watanabe K., Taniguchi T., Moriyama S. (2018). Observation of the quantum valley Hall state in ballistic graphene superlattices. Sci. Adv..

[34] Lee K.W., Lee C.E. (2020). Quantum valley Hall effect in wide-gap semiconductor SiC monolayer. Sci. Rep..

[35] Wang G., Chernikov A., Glazov M.M., Heinz T.F., Marie X., Amand T., Urbaszek B. (2018). Colloquium: Excitons in atomically thin transition metal dichalcogenides. Rev. Mod. Phys..

[36] Nguyen P.V., Teutsch N.C., Wilson N.P., Kahn J., Xia X., Graham A.J., Kandyba V., Giampietri A., Barinov A., Constantinescu G.C. (2019). Visualizing electrostatic gating effects in two-dimensional heterostructures. Nature.

[37] Liu F., Ziffer M.E., Hansen K.R., Wang J., Zhu X. (2019). Direct Determination of Band-Gap Renormalization in the Photoexcited Monolayer *MoS*_2_. Phys. Rev. Lett..

[38] Marino E.C. (2017). Quantum Field Theory Approach to Condensed Matter Physics.

[39] Zhao P.L., Wang A.M., Liu G.Z. (2018). Condition for the emergence of a bulk Fermi arc in disordered Dirac-fermion systems. Phys. Rev. B.

[40] Wang J. (2018). Role of four-fermion interaction and impurity in the states of two-dimensional semi-Dirac materials. J. Phys. Condens. Matter.

[41] Fernández L., Alves V.S., Gomes M., Nascimento L.O., Peña F. (2021). Influence of the four-fermion interactions in a (2+1)D massive electron system. Phys. Rev. D.

[42] Aoyama T., Hayakawa M., Kinoshita T., Nio M. (2012). Tenth-Order QED Contribution to the Electron *g*-2 and an Improved Value of the Fine Structure Constant. Phys. Rev. Lett..

[43] Kurganova E.V., van Elferen H.J., McCollam A., Ponomarenko L.A., Novoselov K.S., Veligura A., van Wees B.J., Maan J.C., Zeitler U. (2011). Spin splitting in graphene studied by means of tilted magnetic-field experiments. Phys. Rev. B.

[44] Song Y.J., Otte A.F., Kuk Y., Hu Y., Torrance D.B., First P.N., de Heer W.A., Min H., Adam S., Stiles M.D. (2010). High-resolution tunnelling spectroscopy of a graphene quartet. Nature.

[45] Hill H.M., Rigosi A.F., Roquelet C., Chernikov A., Berkelbach T.C., Reichman D.R., Hybertsen M.S., Brus L.E., Heinz T.F. (2015). Observation of excitonic Rydberg states in monolayer MoS2 and WS2 by photoluminescence excitation spectroscopy. Nano Lett..

[46] Chernikov A., Berkelbach T.C., Hill H.M., Rigosi A., Li Y., Aslan B., Reichman D.R., Hybertsen M.S., Heinz T.F. (2014). Exciton Binding Energy and Nonhydrogenic Rydberg Series in Monolayer *WS*_2_. Phys. Rev. Lett..

[47] He K., Kumar N., Zhao L., Wang Z., Mak K.F., Zhao H., Shan J. (2014). Tightly Bound Excitons in Monolayer *WSe*_2_. Phys. Rev. Lett..

[48] Marino E.C., Nascimento L.O., Alves V.S., Menezes N., Smith C.M. (2018). Quantum-electrodynamical approach to the exciton spectrum in transition-metal dichalcogenides. 2D Mater..

[49] Keldysh L. (2024). Coulomb interaction in thin semiconductor and semimetal films. Selected Papers of Leonid V Keldysh.

[50] Marino E. (1991). Complete bosonization of the Dirac fermion field in 2 + 1 dimensions. Phys. Lett. B.

[51] Teber S. (2012). Electromagnetic current correlations in reduced quantum electrodynamics. Phys. Rev. D.

[52] Teber S., Kotikov A. (2018). Field theoretic renormalization study of interaction corrections to the universal ac conductivity of graphene. J. High Energy Phys..

[53] Teber S. (2014). Two-loop fermion self-energy and propagator in reduced *QED*_3,2_. Phys. Rev. D.

[54] Guzmán V.M.B., Bashir A., Albino L., Rodríguez-Tzintzun D. (2023). One loop reduced QED for massive fermions within an innovative formalism. Phys. Rev. D.

[55] Magalhães G.C., Alves V.S., Nascimento L.O., Marino E.C. (2021). Bosonization, mass generation, and the pseudo-Chern-Simons action. Phys. Rev. D.

[56] Silva J.D.L., Braga A.N., Pires W.P., Alves V.S., Alves D.T., Marino E.C. (2017). Inhibition of the Fermi velocity renormalization in a graphene sheet by the presence of a conducting plate. Nucl. Phys. B.

[57] Pires W.P., Silva J.D.L., Braga A.N., Alves V.S., Alves D.T., Marino E.C. (2018). Cavity effects on the Fermi velocity renormalization in a graphene sheet. Nucl. Phys. B.

[58] González J., Guinea F., Vozmediano M.A.H. (1999). Marginal-Fermi-liquid behavior from two-dimensional Coulomb interaction. Phys. Rev. B.

[59] Pedrelli D.C., Alves D.T., Alves V.S. (2020). Two-loop photon self-energy in pseudoquantum electrodynamics in the presence of a conducting surface. Phys. Rev. D.

[60] Braga A.N., Pires W.P., Silva J.D.L., Alves D.T., Alves V.S. (2024). Renormalization group functions in two-dimensional massive Dirac-like systems near an interface. Phys. Rev. D.

[61] Vozmediano M.A.H., Guinea F. (2012). Effect of Coulomb interactions on the physical observables of graphene. Phys. Scr..

[62] Caramês L.G.P. (2019). Pseudo-Quantum Electrodynamics in the Presence of Partially Reflective Surfaces. Master’s Thesis.

[63] Gorbar E.V., Gusynin V.P., Parymuda M.R. (2023). Reduced QED with Few Planes and Fermion Gap Generation. Entropy.

